# A meta-analysis of published data on the efficacy and safety of radiotherapy combined with systemic therapy in advanced esophageal carcinoma

**DOI:** 10.3389/fonc.2026.1719295

**Published:** 2026-01-27

**Authors:** Xiaoxi Chen, Rutian Cheng, Shuang Liu, Yu Cao, Lihong Liu, Hua Dong, Xuejiao Ren, Shutang Liu, Xiaoning Li, Chun Han, Lan Wang

**Affiliations:** 1 Department of Radiation Oncology, The Fourth Hospital of Hebei Medical University, Shijiazhuang, China; 2 Anti-Cancer Association of Hebei Province, The Fourth Hospital of Hebei Medical University, Shijiazhuang, China

**Keywords:** advanced esophageal cancer, chemotherapy, immunotherapy, meta-analysis, radiotherapy, systemic therapy

## Abstract

**Introduction:**

Chemoimmunotherapy or chemotherapy-based drug therapy is the preferred treatment for advanced esophageal cancer. However, the prognosis of patients is poor, there is an urgent need to explore more effective combined treatment strategies to improve survival outcomes. In this study, we conducted a meta-analysis to evaluate the efficacy and safety of combined treatment with chemoimmunotherapy or chemotherapy and radiotherapy.

**Methods:**

PubMed, Embase, Web of Science, Cochrane Library and China National Knowledge Infrastructure (CNKI) were searched using a combination of subject terms and free words. Data were extracted from studies that met the inclusion criteria and meta-analyzed using Stata 17.0 software to compare the efficacy and treatment-related adverse toxicities of chemotherapy/chemoimmunotherapy plus radiotherapy with chemotherapy/chemoimmunotherapy alone.

**Results:**

Finally, a total of 12 studies (11 retrospective cohort studies and 1 randomized controlled trial) involving 2,428 patients were included in the analysis, with squamous cell carcinoma accounting for 98.9%. In terms of efficacy, meta-analysis revealed the radiotherapy combined group (RT group) had significantly higher objective response rate (ORR), disease control rate (DCR), progression-free survival (PFS), and overall survival (OS) than those of the non-radiotherapy combined group (NRT group). Stratified analysis showed that for patients with immune checkpoint inhibitors (ICIs)-based systemic treatment and oligometastatic esophageal cancer, the OS and PFS of the RT group were significantly better than those of the NRT group (ICIs ± radiotherapy: Hazard ratio (HR)=0.61, 95%CI:0.48-0.76, P<0.001; HR = 0.60, 95%CI:0.49-0.73, P<0.001, oligometastatic esophageal cancer: HR = 0.73, 95% CI: 0.64-0.84, P<0.001; HR = 0.66, 95% CI: 0.58-0.76, p<0.001). The incidence of grade≥3 hematological toxicity and treatment-related pneumonia in the RT group was higher than that in the NRT group, and there was no significant difference in gastrointestinal adverse reactions between the two groups(2.5%-12.2% vs. 3.4%-13.2%, P = 0.828).

**Conclusions:**

Based on the current data, systemic therapy combined with local radiotherapy may be a better option for advanced esophageal cancer, but the potential risk of higher hematological toxicity and treatment-related pneumonia need to be weighed.

**Systematic Review Registration:**

https://www.crd.york.ac.uk/prospero/, PROSPERO identifier CRD420251101459.

## Introduction

For advanced esophageal cancer, a comprehensive treatment approach based on systemic therapy is emphasized. In the era before immunotherapy, advanced esophageal cancer was primarily treated with chemotherapy, which had a poor prognosis, and the median overall survival (OS) time was often less than 1 year (1–3). And once the disease progressed, there were limited options for second-line drugs, with an efficacy rate of less than 10% (4, 5), posing a challenging problem for clinical treatment. In recent years, immune checkpoint inhibitors (ICIs) had largely changed the treatment pattern of esophageal cancer. With the publication of results from a series of phase III studies, including KEYNOTE-590, CHECKMATE-648, and ESCORT-1st (6–12), chemoimmunotherapy had emerged as the new standard first-line treatment for advanced esophageal cancer. However, even so, data from these studies indicated that the median survival time for patients undergoing first-line chemoimmunotherapy was only 12.4-17.2 months (6–12), which still fallen short of meeting clinical needs. Data from previous retrospective studies (13) indicated that oligoprogression/oligorecurrence (≤2 disease sites) was the most prevalent treatment failure mode (70.5%) after immunotherapy for gastrointestinal system tumors, in which lymph nodes were the most common site of progression (58.4%), and the prognosis of patients with oligoprogression was significantly better than that of patients with multiple progression (38.5m vs. 14.0m; HR = 0.37; 95% CI, 0.18-0.74; P < 0.001). Hence, for advanced esophageal cancer, we posit that combining radiotherapy with chemo-immunotherapy offers substantial opportunities for exploration and potential benefit value. Based on this, we conducted this meta-analysis to investigate the survival benefit and safety of combining radiotherapy with previous standard systemic therapy, particularly with systemic therapy based on immune checkpoint inhibitors (ICIs). As China has the highest incidence of esophageal cancer in the world, this study particularly focuses on reports and data from China (i.e. China National Knowledge Infrastructure (CNKI)).

## Materials and methods

### Literature search

The study protocol was registered on PROSPERO(CRD420251101459). By conducting a systematic search of PubMed, Embase, Web of Science, the Cochrane Library, and CNKI using both controlled vocabulary and free-text terms up to April 2025, the English search terms included “Esophageal Cancer”, “Esophageal Neoplasm”, “Radiotherapy”, “Immunotherapy”, and “Chemotherapy,”. Following retrieval of the literature, titles and abstracts were meticulously reviewed with full-text examination as necessary. Subsequently, in accordance with predefined inclusion and exclusion criteria, relevant studies were systematically screened and summarized as presented in **Table 1**.

**Table 1:** Characteristics of included studies.

Reference	Pathology	Case(RT/NRT)	Treatment		Research	NOS/Jadad
			RT	NRT		
Ma et al. (17) 2022	SCC/Others(71/9)	80(35/45)	ICIs+CRT(5-FU/PTX+DDP)	ICIs + CT(5-FU/PTX)+DDP)	RCS	6
Wu et al. (18) 2022	SCC(127)	127(40/87)	30-60Gy RT +ICIs+CT	ICIs+CT	RCS	7
Lyu et al. (19) 2018	SCC(141)	141(55/86)	50.0-66.0Gy RT+CT(5-FU/PTX+DDP)	CT(5-FU/PTX +DDP)	RCS	7
Chen et al. (20) 2019	SCC/EAC(446/15)	461(196/265)	45-50Gy RT+CT(PTX +DDP)	CT(PTX +DDP)	RCS	8
Shi et al. (21) 2022	SCC(532)	532(240/292)	45-50GyRT+CT(5-FU/DTX +DDP)	CT(5-FU/DTX + DDP)	RCS	8
Li et al. (22) 2022	SCC(194)	194(101/93)	≥50.4Gy RT + CT(based on DDP)	CT(based on DDP)	RCS	8
Zhang et al. (23) 2008	SCC(44)	44(21/23)	50-60Gy RT+CT(PTX +DDP)	CT(PTX +DDP)	RCS	6
Zhao et al. (24) 2023	SCC(198)	198(136/62)	30-60Gy RT+ICIs+CT	ICIs/ICIs + CT	RCS	7
Wang et al. (25) 2022	SCC/EAC(48/2)	50(24/26)	RT+(sintilimab /sintilimab+CT/sintilimab+antiangiogenic)	sintilimab/sintilimab+CT/sintilimab+antiangiogenic therapy	RCS	7
Li et al. (26) 2021	SCC(247)	247(191/56)	≤ 70 Gy RT+CT	CT	RCS	7
Liu et al. (27) 2023	SCC(104)	104(53/51)	SABR/IMRT/Surgery/Heat therapy +CT/ICIs+CT	CT/ICIs+CT(DDP+PTX/DTX/CPT-11)	RCT	5*
Chen et al. (28) 2024	SCC(250)	250(125/:125)	RT+CT(platinum or fluoropyrimidine-based)+ ICIs	CT(platinum or fluoropyrimidine-based)+ ICIs	RCS	8

SCC, Squamous Cell Carcinoma; EAC, Esophageal Adenocarcinoma; RT, radiotherapy; CT, chemotherapy; ICIs, immune checkpoint inhibitors; DDP, cisplatin; 5-fu, 5-fluorouracil; DTX, docetaxel; PTX, paclitaxel; CPT-11, irinotecan; sintilimab, sindilizumab; Gy, Gray; RCT, randomized controlled study; RCS, retrospective cohort study; NOS, Newcastle-Ottawa Quality Assessment Scale; *, Application of the Jadad Scoring System.

### Literature inclusion criteria

The inclusion criteria were as follows: (1) Randomized controlled trials (RCTs), retrospective or prospective cohort studies (RCSs); (2) studies comparing the efficacy and safety of combined local and systemic treatment with pure systemic treatment for advanced or recurrent esophageal cancer; (3) the observational indicators of the study are focused on treatment effects and toxicity of both regimens; (4) all study subjects must be histopathologically confirmed as having esophageal cancer, excluding gender, age, or clinical staging-based studies, with Tumor Node Metastasis (TNM) staging defined as advanced; (5) at least one or more outcome indicators requiring analysis should be included in the study.

### Exclusion criteria

The exclusion criteria were as follows: (1) Co-study of other malignant tumors or concurrent diseases; (2) inclusion of patients in the early stages of cancer, studies involving neoadjuvant or adjuvant radiotherapy or chemotherapy; (3) articles lacking the requisite data for this study or with incomplete data; (4) studies with sample sizes <40; (5) review-type studies, systematic reviews, conference abstracts, and case reports.

### Intervention measures

The Radiotherapy combined group (RT group) was treated with chemotherapy or immunotherapy or chemo-immunotherapy combined with radiotherapy. The non-radiotherapy combined group (NRT group) was treated with chemotherapy, immunotherapy or chemo-immunotherapy alone.

### Observation indices and reference variables

#### Safety

The main adverse reactions caused by the treatment are hematologic toxicity, gastrointestinal reactions, and radiation pneumonitis. This study primarily considered grade 3 and above adverse reactions. Grade 3 and above adverse reactions may require treatment interruption, affecting treatment efficacy and impacting patients’ quality of life. The short-term therapeutic effects were assessed based on the WHO RECIST criteria. Tumor response was categorized as complete response (CR), partial response (PR), stable disease (SD), or progressive disease (PD). Objective response rate (ORR) = complete response (CR) + partial response (PR), disease control rate (DCR) = CR + PR + SD. Survival indicators included overall survival (OS) and progression-free survival (PFS).

### Quality evaluation

The retrospective cohort studies were evaluated for quality using the Newcastle-Ottawa Scale (NOS) (14, 15), which encompasses three domains: selection of patients, comparability between study groups, and outcome assessment. Two researchers independently appraised the quality of each cohort study and assigned scores, with a maximum score of 9 points. Studies achieving a score of 6 or higher were deemed to be of high quality and met the criteria for inclusion in the analysis. The randomized controlled trials were assessed using the modified Jadad quality score (16), which considers randomization, blinding, and documentation of withdrawals and losses to follow-up. A maximum score of 7 points was possible, with studies scoring 1–3 classified as low quality and those scoring 4–7 as high quality.

### Data extraction

Two independent evaluators extracted the study data, and discrepancies were resolved through discussion or consultation with a third party (the supervising teacher) after cross-checking. The basic information extracted included the sample size, pathological type, chemotherapy regimen, radiotherapy dose, study type, short-term treatment efficacy, overall survival rate (OS), progression-free survival rate (PFS), and grade 3 or higher toxicity. For studies that provided Kaplan-Meier survival curves instead of raw values, we utilized Engauge-Digitizer software to extract information from the Kaplan-Meier curves.

### Statistical analysis

The statistical analysis of safety data (grade 3 or higher toxic reactions) and short-term efficacy (ORR, DCR) in the treatment of esophageal cancer was performed using Stata 17.0. The relative risk (RR) was chosen as the effect measure, and the 95% confidence interval (CI) was utilized to indicate the magnitude of the effect. Heterogeneity between trial groups was assessed using the chi-square test; if P≥0.05 and I^2^ ≤ 50%, it indicated good heterogeneity between studies, and a fixed-effect model (FEM) was employed for analysis. If P<0.05 and I^2^>50%, indicating substantial heterogeneity between studies, sensitivity analysis was conducted to evaluate sources of heterogeneity and assess result robustness. If heterogeneity persisted after removal, a random-effects model (REM) was used. Using Stata 17.0, the risk ratio (hazard ratio, HR) was utilized to assess the OS and PFS in the included studies. The effect size was presented as a 95% CI. Heterogeneity between trial groups was assessed using the chi-square test. When P≥0.05 and I^2^ ≤ 50%, it indicated well-homogeneous studies, and analysis employed a FEM. Conversely, if P<0.05 and I^2^>50%, significant heterogeneity existed, leading to utilization of a REM. Furthermore, Begg and Egger tests were conducted to evaluate publication bias; non-significant publication bias was indicated by P>0.05. In cases where publication bias was present (P<0.05), results were evaluated for robustness using the trim-and-fill method.

## Results

### Basic information on the literature retrieval

**Figure 1:**
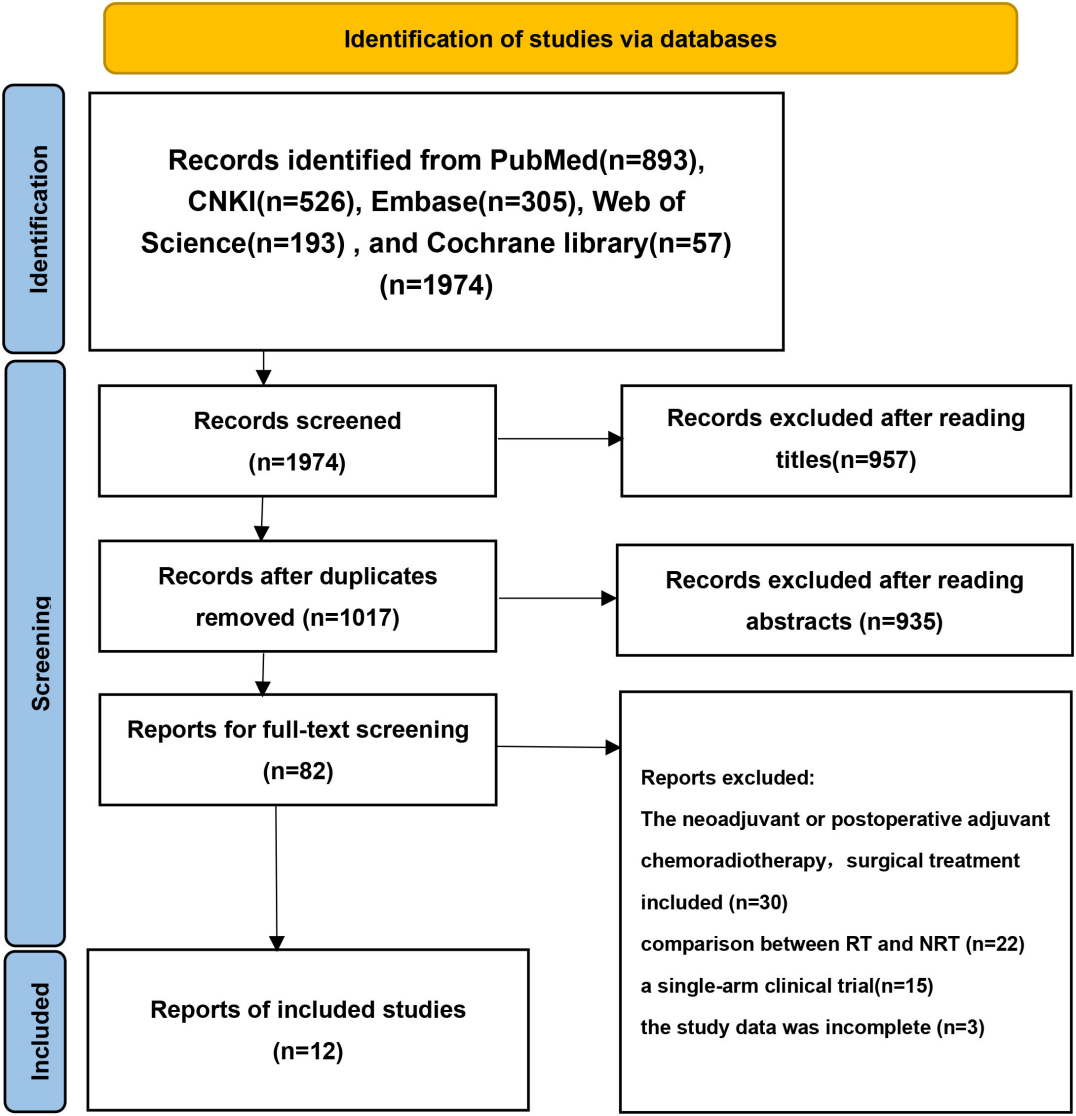
Flow diagram showing the selection process for included studies.

Following the search strategy, 2,740 pertinent studies were initially retrieved from the database using Endnote X9 software. After excluding duplicate studies, 1,017 remained. Upon reviewing the titles and abstracts and eliminating those that clearly did not meet the inclusion criteria, 81 studies were retained. Subsequent meticulous examination of the full texts led to the final inclusion of 12 studies (17–28), comprising 11 retrospective cohort studies and 1 randomized controlled trial with a total enrollment of 2,428 patients. Among them, 1,217 (50.1%) were in the RT group and 1,211 (49.9%) were in the NRT group. The specific selection process is illustrated in **Figure 1**.

### Basic characteristics of the included studies

The final 12 studies included in the systematic review were all conducted in China, encompassing 2402 cases (98.9%) of squamous cell carcinoma, 17 cases (0.7%) of adenocarcinoma, and 9 cases (0.4%) of indeterminate pathological type. Among these studies, six studies (19– 23, 26) employed chemotherapy combined with radiotherapy as the observational regimen, while five studies (17, 18, 24, 27, 28) utilized chemoimmunotherapy alongside radiotherapy. Additionally, two studies (25, 28) implemented immunotherapy in conjunction with radiotherapy and three studies (21, 21, 27) reported survival data for oligometastatic esophageal cancer. The fundamental characteristics of these studies are detailed in **Table 1**. The retrospective studies’ quality was assessed using the NOS which assigns scores represented by stars with a maximum score of nine points, and the results are presented in **Table 2**. Furthermore, one randomized controlled trial underwent evaluation using the modified Jadad quality score and received a score of five.

**Table 2:** Quality evaluation of retrospective studies.

Reference	Object selection				Comparability			Result		Total score
	1	2	3	4	5	6	7	8	9	
Ma et al. (17) 2022	—	☆	☆	☆	—	☆	—	☆	☆	6
Wu et al. (18) 2022	☆	☆	☆	☆	☆	☆	—	—	☆	7
Lyu et al. (19) 2018	☆	☆	☆	☆	—	☆	—	☆	☆	7
Chen et al. (20) 2019	—	☆	☆	☆	☆	☆	☆	☆	☆	8
Shi et al. (21) 2022	☆	☆	☆	☆	☆	☆	—	☆	☆	8
Li et al. (22) 2022	☆	☆	☆	☆	☆	☆	—	☆	☆	8
Zhang et al. (23) 2008	—	☆	☆	☆	—	☆	—	☆	☆	6
Zhao et al. (24) 2023	☆	☆	☆	☆	—	☆	☆	—	☆	7
Wang et al. (25) 2022	☆	☆	—	☆	☆	☆	☆	—	☆	7
Li et al. (26) 2021	☆	☆	☆	☆	—	☆	—	☆	☆	7
Chen et al. (28) 2024	☆	☆	☆	☆	☆	☆	—	☆	☆	8

—: 0 points; ☆: 1 point; Item 1: Representatives of the exposed cohort; Item 2: Selection method of the non-exposed cohort; Item 3: Method for ascertaining exposure; Item 4: Outcome of interest was not present at the start of the study; Item 5: Comparability between the study and control groups; Item 6: Whether the study design is free of bias; Item 7: Adequacy of outcome assessment; Item 8: Follow-up duration was sufficiently long after the occurrence of the outcome; Item 9: Completeness of follow-up.

### Comparison of the short-term therapeutic effects of the RT and NRT group

**Figure 2:**
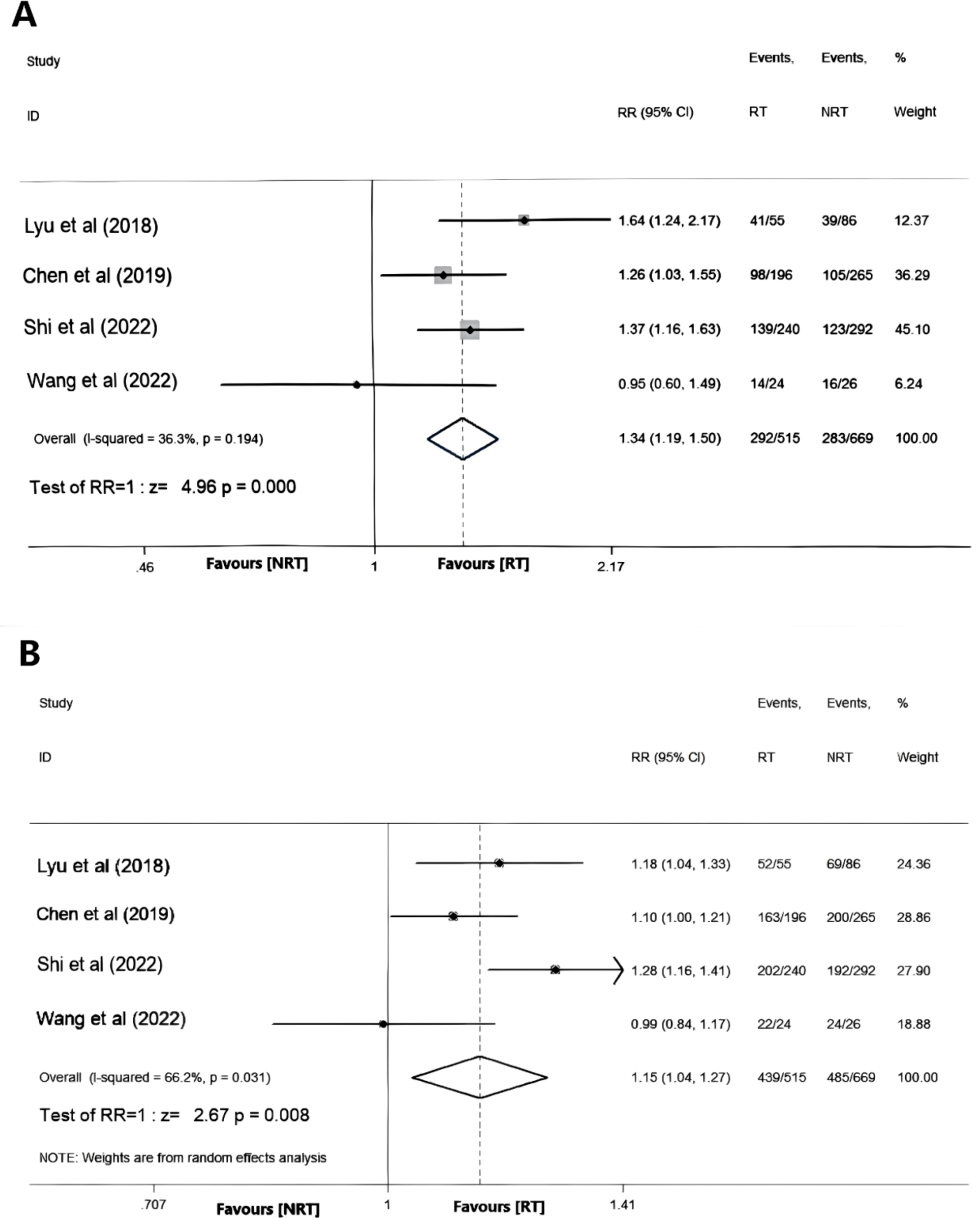
Forest plot of objective response rate **(A)** and disease control rate **(B)**.

Five studies reported the ORR and DCR results respectively, and statistics showed that there was significant heterogeneity among the studies (ORR: I²=70.3%, P< 0.001; DCR: I²= 88.7%, P < 0.001). A sensitivity analysis identified that the study by Zhao et al. (24) had a substantial impact on the overall findings. Upon exclusion of this study and re-evaluation for heterogeneity, it was determined that the remaining four studies exhibited no significant heterogeneity (I² = 36.3%, P = 0.194). Subsequently, a fixed-effects model was employed for meta-analysis, revealing a statistically significant difference in ORR between the RT and NRT groups, with higher rates observed in the former (RR = 1.34, 95%CI: 1.19-1.50, P<0.001), as depicted in **Figure 2A**.

For DCR, after excluding the study by Zhao et al. (24), the remaining four studies still had significant heterogeneity(I²=66.2%,P=0.031). Utilizing a random effects model for meta-analysis demonstrated significantly higher DCR rates in the RT group compared to the NRT group (RR = 1.15, 95%CI: 1.04-1.27, P = 0.008), as illustrated in **Figure 2B**.

### Comparison of the OS and PFS rates between the RT and NRT group

**Figure 3:**
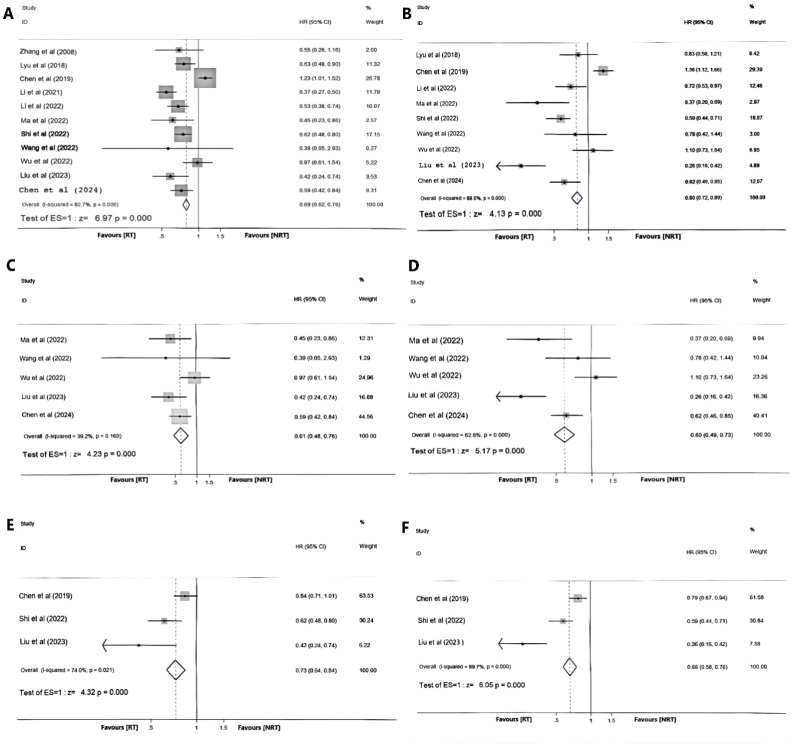
Forest plot of OS **(A)** and PFS **(B)** in the RT group and NRT group; Forest plot of OS **(C)** and PFS **(D)** for the immunotherapy-based drug regimens in the RT group and NRT group; Forest plot of OS **(E)** and PFS **(F)** for oligometastatic subgroup.

The OS data for this study were derived from 11 studies (17– 23, 25–27 ,28), while the PFS data were obtained from 9 studies (17–22, 25, 27, 28). HR values were extracted from these studies. In cases where only Kaplan-Meier survival curves were available, specific OS and PFS data points were extracted from the curves using Engauge-Digitizer software to calculate the HR values as detailed in **Table 3**. Upon examination, high inter-study heterogeneity was observed for both OS and PFS (I^2^ >50%, P<0.05), with study by Zhao et al. (24) significantly impacting the results. Following exclusion of this study, a subsequent heterogeneity analysis was conducted utilizing a random effects model. The effect measure selected was HR, and meta-analysis results indicated that both OS and PFS in the RT group exhibited significant improvement compared to those in the NRT group (HR = 0.69, 95%CI:0.62-0.76, P<0.001; H:I^2^ = 82.7%, p <0.001;HR =0.80, 95%CI:0.72-0.89, p<0.001; H:I^2^ = 88.0%, p <0.001). The forest plot is depicted in **Figures 3A, B**.

**Table 3:** Short-term therapeutic effect, OS, PFS and AEs in RT group and NRT group.

Index	Effect model	Test value	Range(%)		RR^*^/HR^#^	95%CI	P value
			RT group	NRT group			
Short term therapeutic effect							
ORR	Fixed effects model	I^2^ = 36.3%, P = 0.194	50.0-74.5	27.4-61.5	1.34^*^	1.19-1.50	<0.001
DCR	Randomeffects model	I^2^ = 66.2%, P = 0.031	83.2-94.5	65.8-96.8	1.15^*^	1.04-1.27	0.008
OS							
1-y OS rate	Random effects model	I^2^ = 82.7%, P<0.001	28.0-94.3	4.0-80.4	0.69^#^	0.62-0.76	<0.001
2-y OS rate			14.0-56.6	2.0-42.7			
3-y OS rate			2.9-50.7	0-42.7			
PFS							
1-y PFS rate	Random effects model	I^2^ = 88.0%, P<0.001	25.0-64.7	8.9-54.8	0.80^#^	0.72-0.89	<0.001
2-y PFS rate			8.6-38.2	0-36.7			
3-y PFS rate			0.5-34.5	0-32.3			
OS of immunotherapy							
1-y OS rate	Random effects model	I^2^ = 39.2%, P = 0.160	47.5-94.3	37.8-80.4	0.61^#^	0.48-0.76	<0.001
2-y OS rate			22.5-56.6	4.4-41.9			
3-y OS rate			2.9-50.7	0-41.9			
PFS of immunotherapy							
1-y PFS rate	Random effects model	I^2^ = 82.8%, P<0.001	25.0-64.7	8.9-54.8	0.60^#^	0.49-0.73	<0.001
2-y PFS rate			8.6-38.2	2.2-35.4			
OS of oligometastatic							
1-y OS rate	Random effects model	I^2^ = 74.0%, P = 0.021	66.8-94.3	55.5-80.4	0.73**^#^**	0.63-0.84	<0.001
2-y OS rate			16.3-56.6	12.4-29.4			
3-y OS rate			2.6-18.9	1.5-4.5			
PFS of oligometastatic							
1-y PFS rate	Random effects model	I^2^ = 89.7%, P<0.001	25.0-62.2	19.6-29.4	0.66^#^	0.58-0.76	<0.001
2-y PFS rate			2.0-30.2	0.7-7.8			
3-y PFS rate			0.5-9.4	0-1.4			
AEs(≥3 grade)							
Hematological toxicity	Random effects model	I^2^ = 47.2%, P = 0.078	9.5-41.8	4.3-35.3	1.22^*^	1.03-1.45	0.024
Gastrointestinal reactions	Fixed effects model	I^2^ = 0.0%, P = 0.859	2.5-12.2	3.4-13.2	0.96^*^	0.69-1.34	0.828
Pneumonitis	Fixed effects model	I^2^ = 0.0%, P = 0.623	0-7.7	0-2.2	2.96^*^	1.44-6.07	0.003

RT group, Radiotherapy combined group; NRT group, Non-radiotherapy combined group; OR, Odds Ratio; HR, Hazard ratio; CI, Confidence Intervals; ORR, Objective remission rate; DCR, Disease control rate; OS, Overall survival; PFS, Progression-Free Survival; AEs, Adverse Events.

### Survival analysis of immunotherapy-based drug regimens with/without radiotherapy

To further investigate the potential benefits of combining local radiation therapy with immunotherapy-based drug regimens for patients, we conducted a stratified analysis of 6 studies on immunotherapy (17, 18, 24, 25, 27, 28) based on the control group intervention. Among these studies, OS and PFS data were reported. After excluding the high heterogeneity group (Zhao et al. (24)), a total of 661 patients were included in the analysis. There was no obvious heterogeneity among the studies regarding OS (I²=39.2%, P = 0.160). Using a fixed-effects model for analysis, the results indicated that OS was significantly better in the RT group compared to the NRT group (HR = 0.61, 95%CI:0.48-0.76, P<0.001). There was significant heterogeneity among the studies regarding PFS. A random effects model analysis also showed that the RT group had a better PFS (HR = 0.60, 95%CI:0.49-0.73, P<0.001; H:I^2^ = 82.8%, p<0.001) (**Figures 3C, D**).

### Survival analysis of oligometastatic esophageal carcinoma

Among the included studies, 3 studies (20, 21, 27) analyzed the benefit of combined treatment strategies for oligometastatic esophageal cancer; all 3 studies provided OS and PFS data and included 1097 patients. Owing to substantial inter-study heterogeneity (I² > 50%, P < 0.05), a random effects model was employed. The meta-analysis findings revealed statistically significant differences in both OS and PFS between the RT group and NRT group (HR = 0.73, 95% CI: 0.64-0.84, P < 0.001; H: I^2^ = 74.0%, P = 0.021; HR = 0.66, 95% CI: 0.58-0.76, P <0.001; H: I^2^ = 89.7%, P <0.001), with RT demonstrating superiority over NRT in reducing mortality and disease progression risks for patients receiving combined radiation therapy compared to those undergoing solely drug therapy as depicted in **Figures 3E, F**.

### Comparison of the toxicities between the RT and NRT group

In the included studies, eight trials (18–23, 27, 28) documented hematological toxicity in the form of leukopenia, while five trials (18–21, 23) reported gastrointestinal reactions, predominantly nausea and vomiting. Additionally, four trials (17, 18, 20, 27) recorded treatment-related pneumonia. All these studies were evaluated based on the critical threshold of ≥3 grade toxicity.

**Figure 4:**
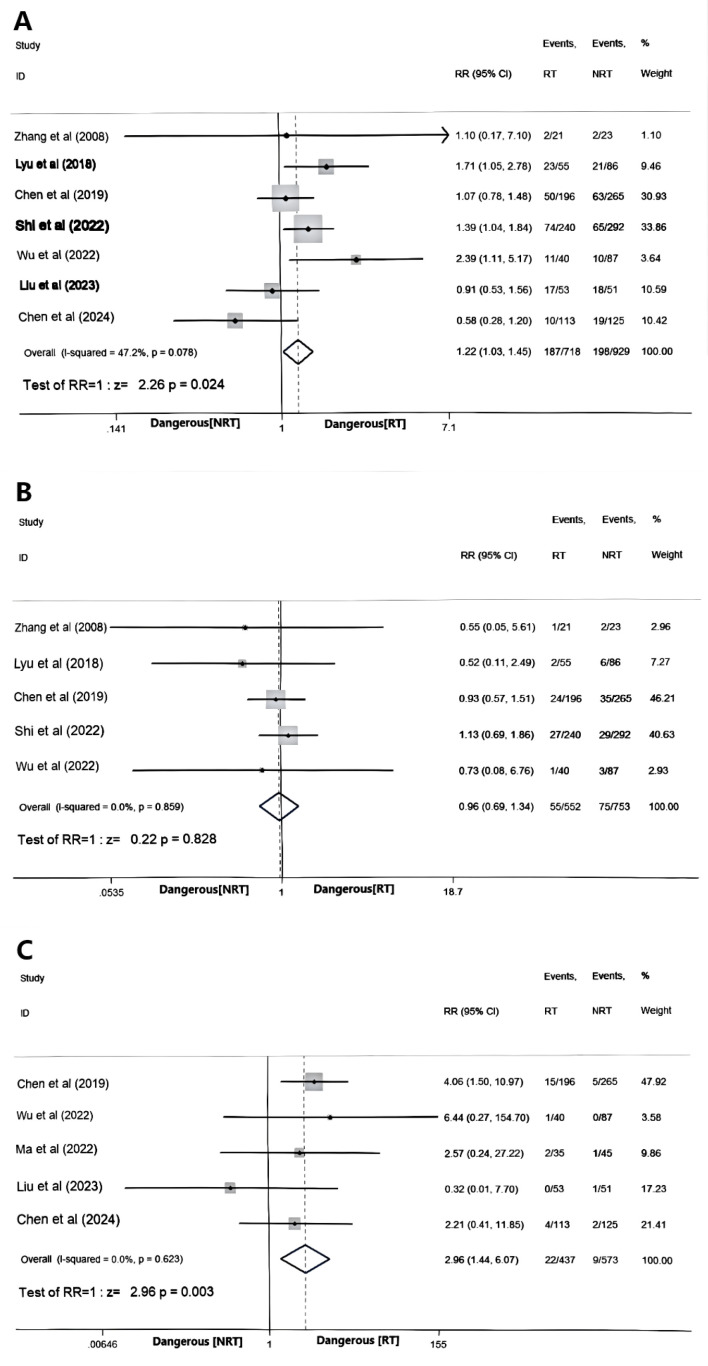
Forest plot of hematological toxicity **(A)**, gastrointestinal toxicity **(B)**, and treatment-associated pneumonia **(C)**.

In terms of hematological toxicity, there was heterogeneity among the 8 studies (I^2^ = 63.1%, P = 0.008), and a sensitivity analysis was conducted using the one-by-one exclusion method. After excluding Li et al. (22), the heterogeneity decreased (I^2^ = 47.2%, P = 0.078). A fixed effects model was employed for analysis, revealing that the hematological toxicity of the RT group exceeded that of the NRT group (RR = 1.22, 95%CI:1.03-1.45, P = 0.024). Regarding gastrointestinal toxicity and treatment-related pneumonia, no significant heterogeneity among the 5 studies was observed (I^2^ = 0.0%, P = 0.859; I^2^ = 0.0%, P = 0.623). A fixed effects model was utilized for analysis, demonstrating that the gastrointestinal toxicity of the RT group did not significantly differ from that of the NRT group (RR = 0.96, 95%CI:0.69-1.34, P = 0.828), but the incidence of grade ≥3 pneumonia in the RT group exceeded that in the NRT group (RR = 2.96, 95%CI:1.44-6.07, P = 0.003), as depicted in **Figures 4A–C**.

### Sensitivity and bias analyses

In the analysis of ORR and hematological toxicity, after excluding studies with a greater impact on the results through sensitivity analysis, the meta-analysis results remained basically unchanged, suggesting that the model is robust. The results showed that the Begg test and Egger test value P > 0.05 for each study indicated that there was no publication bias (**Table 4**).

**Table 4:** Evaluation of publication bias in included studies (*P* values of Begg’s and Egger’s tests).

Items	Begg’s test		Egger’s test	
	z	P	t	P
ORR	0.34	0.734	-0.56	0.630
DCR	0.34	0.734	-0.84	0.489
OS	0.00	1.000	-1.45	0.181
PFS	0.52	0.602	-1.76	0.121

The P-values for both Begg’s and Egger’s tests > 0.05, indicating no evidence of publication bias.

## Discussion

Comprehensive treatment with systemic treatment as the main approach is emphasized for advanced esophageal cancer. In the era of immunotherapy, chemoimmunotherapy combined treatment has emerged as the standard first-line approach for recurrent/metastatic esophageal cancer (1A). However, despite this, the ORR rate of first-line chemoimmunotherapy is only 45%-72.1%, with a median PFS of 5.7-7.3 months (6–12) and a median overall survival of 12.4–17 months, indicating that approximately 30% of advanced cancer patients exhibit initial resistance, and most patients will experience disease progression within 5.7-7.3 months. In fact, for newly diagnosed advanced esophageal cancer, nearly half of the patients still have few metastatic lesions (≤2) (29). For patients with advanced disease who have undergone treatment, the oligoprogression(≤2 metastatic lesions) is the main pattern of progression (122/173, 70.5%), predominantly involving lymph nodes (58.4%) (13). For the above-mentioned subgroups, there is undoubtedly room for exploration and potential benefit value in combining local treatment, especially radiotherapy, on the basis of systemic treatment. However, due to limited evidence, radiotherapy has not yet established its status in the treatment of advanced esophageal cancer in current authoritative guidelines. Against this backdrop, we undertook this meta-analysis to assess the effectiveness and safety of combining local radiotherapy with systemic therapy in comparison to systemic therapy alone for advanced esophageal cancer.

After conducting a comprehensive literature search and selection, we identified 12 studies, all conducted by research teams from China. The case samples included in these studies comprised 98.9% squamous cell carcinoma, enhancing the representativeness of this meta-analysis for esophageal squamous cell carcinoma outcomes in Asia. From the closely monitored efficacy endpoints of OS and PFS, the 1-, 2-, and 3-year OS rates reported in the RT group ranged from 28% to 94.3%, 14% to 56.6%, and 5.2% to 50.7%, respectively, while those in the NRT group ranged from 4% to 80.4%, 2% to 42.7%, and from nil to a maximum of up to 42.7%. The annual OS rates in the former consistently exceeded those in the latter. Furthermore, the respective 1-,2-, and 3-year PFS rates reported for the RT group were within 25%-64.7%, 8.6%-38.2%, and 0.5%-34.5% whereas for the NRT group they were within 8.9%-54.8%, 0-36.7%, and 0-32.3%. The results of the meta-analysis showed that the differences in OS and PFS between the two groups were statistically significant, suggesting that combined radiotherapy with systemic treatment can bring benefits to patients in terms of OS and PFS.

To further explore the therapeutic potential of radiotherapy in the context of immunotherapy for recurrent/metastatic esophageal cancer, we conducted a stratified analysis of six studies that utilized chemotherapy combined with immunotherapy or immunotherapy alone as systemic treatment regimens. These findings suggest that combined radiotherapy continues to yield more optimized survival outcomes within systemic treatments based on ICIs. In the sole prospective randomized controlled trial (27) included in this study, 38% (20 cases) and 45% (23 cases) of patients in respective groups opted for ICIs-based systemic treatment regimens, resulting in median PFS durations of 18.0 months and 9.6 months, respectively (P = 0.044). The RT group exhibited an extension of median time to progression by 8.4 months compared to the NRT group, representing a clinically meaningful PFS benefit. Consequently, we assert that for recurrent/metastatic esophageal cancer, integrating local radiotherapy with chemoimmunotherapy holds great promise as a comprehensive treatment modality likely to confer additional survival benefits for patients.

When exploring the therapeutic benefits of local radiotherapy in advanced esophageal cancer, a very important issue is the screening of the advantageous population. Based on previous reports, oligometastatic/oligoprogressive esophageal cancer is the most likely indication population (27). For instance, as demonstrated in Wu et al. (18), while the addition of radiotherapy to immunotherapy did not yield a significant PFS advantage for the entire cohort of recurrent and metastatic patients (5.45 months vs. 4.60 months, P = 0.660). However, for patients with local regional recurrence, combined radiotherapy can increase the median OS of patients from 7.69 months to 19.48 months, extending the OS time by nearly 1 year, which is a very significant survival benefit (P = 0.026). In our meta-analysis, three cohort studies specifically focused on oligometastatic esophageal cancer. Although the definitions of “oligometastatic” varied among the reports, all three studies demonstrated that systemic therapy combined with local radiotherapy improved OS and PFS, indicating that patients with limited metastatic sites in advanced esophageal cancer may benefit from systemic therapy combined with local radiotherapy and warrant further prospective studies to explore this.

In terms of safety, the meta-analysis results indicate that combined treatment does not lead to an increased incidence of gastrointestinal reactions in patients. The primary risks for adverse events stem from hematological toxicity and treatment-related pneumonia. Among the four studies, the incidence of ≥3 grade pneumonia ranged from 0-7.7% in the RT group and 0-2.2% in the NRT group, with a significantly higher occurrence in the former compared to the latter. However, all reported incidences of ≥3 grade pneumonia fell within clinically acceptable ranges across these studies. Nevertheless, to avoid serious adverse events, proactive monitoring and assessment of risk factors should be conducted prior to combined treatment to predict or mitigate complications during the course of treatment.

The study’s limitations include the retrospective nature of eleven out of twelve selected studies, potentially leading to selection bias between the RT and NRT groups and unbalanced confounding factors. Additionally, variations in chemotherapy regimens, radiotherapy doses, and immune drugs across different studies may impact the reliability of meta-analysis results. Furthermore, all studies were from China, and the meta-analysis may be more representative of the results of Asian patients with advanced esophageal squamous cell cancer (ESCC). Moreover, the advanced esophageal cancer population is highly heterogeneous, and systemic treatment combined with local radiotherapy may not benefit all patients. The screening of the indicated population still needs further discussion.

## Conclusions

Based on the current data, systemic therapy combined with local radiotherapy may be a better option for advanced esophageal cancer, but the potential risk of higher hematological toxicity and treatment-related pneumonia need to be weighed. This finding warrants further evaluation in prospective, large-sample studies.

## Data Availability

The original contributions presented in the study are included in the article/supplementary material. Further inquiries can be directed to the corresponding authors.

## Glossary

CNKI: China National Knowledge Infrastructure
ORR: Objective Response Rate
DCR: Disease Control Rate
PFS: Progression-Free Survival
OS: Overall Survival
ICIs: Immune Checkpoint Inhibitors
HR: Hazard Ratio
RCTs: Randomized Controlled Trials
RCSs: Retrospective Cohort Studies
TNM: Tumor Node Metastasis
CR: Complete Response
PR: Partial Response
SD: Stable Disease
PD: Progressive Disease
NOS: Newcastle-Ottawa Scale
RR: Relative Risk
CI: Confidence Interval
FEM: Fixed-Effect Model
REM: Random-Effects Model
ESCC: Esophageal Squamous Cell Cancer
OR: Odds Ratio
AEs: Adverse Events
SCC: Squamous Cell Carcinoma
EAC: Esophageal Adenocarcinoma
RT: Radiotherapy
CT: Chemotherapy
DDP: Cisplatin
5-fu: 5-fluorouracil
DTX: Docetaxel
PTX: Paclitaxel
CPT-11: Irinotecan
Gy: Gray
